# Nickel-Embedded Carbon Materials Derived from Wheat Flour for Li-Ion Storage

**DOI:** 10.3390/ma13204611

**Published:** 2020-10-16

**Authors:** Wen Ding, Xiaozhong Wu, Yanyan Li, Shuo Wang, Shuping Zhuo

**Affiliations:** School of Chemistry and Chemical Engineering, Shandong University of Technology, Zibo 255049, China; berthading@126.com (W.D.); 18369904378@163.com (Y.L.); wangshuo9@msn.cn (S.W.)

**Keywords:** biomass-based carbon, nickel nanoparticles, lithium-ion batteries, anode material

## Abstract

The biomass-based carbons anode materials have drawn significant attention because of admirable electrochemical performance on account of their nontoxicity and abundance resources. Herein, a novel type of nickel-embedded carbon material (nickel@carbon) is prepared by carbonizing the dough which is synthesized by mixing wheat flour and nickel nitrate as anode material in lithium-ion batteries. In the course of the carbonization process, the wheat flour is employed as a carbon precursor, while the nickel nitrate is introduced as both a graphitization catalyst and a pore-forming agent. The in situ formed Ni nanoparticles play a crucial role in catalyzing graphitization and regulating the carbon nanocrystalline structure. Mainly owing to the graphite-like carbon microcrystalline structure and the microporosity structure, the NC-600 sample exhibits a favorable reversible capacity (700.8 mAh g^−1^ at 0.1 A g^−1^ after 200 cycles), good rate performance (51.3 mAh g^−1^ at 20 A g^−1^), and long-cycling durability (257.25 mAh g^−1^ at 1 A g^−1^ after 800 cycles). Hence, this work proposes a promising inexpensive and highly sustainable biomass-based carbon anode material with superior electrochemical properties in LIBs.

## 1. Introduction

In recent years, with the pollution of the environment and consumption of energy, renewable energy technologies have attracted worldwide attention. The development of efficient energy storage equipment, such as supercapacitors and rechargeable batteries, has been a focused topic now. Lithium-ion batteries (LIBs) occupy important status in the automotive and portable electronic devices such as wearable microelectronic devices and electric transportation on account of their outstanding properties. In the past years, considerable studies have been proposed around the promising battery anode materials [1,2,3,4,5,6].

Carbon materials as typical anode materials have been investigated proverbially nowadays. Owing to the long durability, excellent specific capacity, and superior rate abilities, the hard carbons have been emerging as promising anode materials. Among these, the biomass-based carbon materials have been extensively studied as anode materials in rechargeable batteries for their nontoxicity and abundance resources in the past decades. The carbon materials derived from various biomass sources [7,8,9], such as rice husk [10], starch [11,12,13], nut shells [14,15,16,17], fruit peels [18,19], flour [20,21,22,23] as electrode materials have exhibited superior electrochemistry properties. For example, the spherical hard carbon derived from potato starch gave an invertible capacity of about 531 mAh g^−1^ at 0.1C for LIBs [24]. The orange peel-derived hard carbon displayed the capacity of 301 mAh g^−1^ at 1 A g^−1^ for LIBs [18]. Sweet potato-derived carbon achieved a capacity of 965 mAh g^−1^ at 0.1 A g^−1^ for LIBs [13]. Zhou reported highly graphitic carbon nanosheets derived from the wheat stalk with a high capacity of 502 mAh g^−1^ at 0.1C for LIBs [25]. However, to develop biomass-based carbon anode, materials with high specific capacity and superior rate capacity are still in great need.

Wheat flour is a powder made from the grinding of wheat, mainly composed of starch, gluten, and a small amount of fat. Many studies have showed wheat flour as a promising precursor for carbon electrode materials because of its extensive sources, less toxic effects, high yield, and high carbon content [26]. Yu reported an environmental flour-derived hierarchical nitrogen-doped porous carbon/polyaniline (HPC/PANI) electrode, which displayed a splendid specific capacitance of 1080 F g^−1^ at 1 A g^−1^ [27]. The carbon originated from wheat flour delivered a good specific capacity of 390 mAh g^−1^ at 1C with a good rate and cycle performance for LIBs [28]. The wheat flour-based carbon materials have been used as various kinds of electrode materials for supercapacitors and other batteries recently [27,29,30,31,32]. Recently, metal and metallic oxide encapsulated into porous carbon is considered to be a simple method to obtain composite with good electrochemical property, such as Sb/C composite [33], MnO/metal/carbon nanohybrid [34], CuO/C [35].

In this study, we successfully synthesized a novel hard carbon embedded with nickel nanoparticles (nickel@carbon, denoted as NC-600 and NC-800) by carbonizing the dough which is prepared by mixing wheat flour and the nickel nitrate. In this process, the wheat flour is employed as a carbon precursor, while the nickel nitrate is introduced as both a pore-forming agent and a graphitization catalyst. The in situ formed Ni nanoparticles in the carbonization process, play an important role in the catalyzed graphitization, and regulate the carbon nanocrystalline structure. Owning to the graphite-like carbon microcrystalline structure, the carbon microporosity structure, and the fast charge transfer, the NC-600 sample exhibits a superior specific capacity (700.8 mAh g^−1^ at 0.1 A g^−1^ after 200 cycles), good performance rate (51.3 mAh g^−1^ at 20 A g^−1^), and long-cycling durability (257.25 mAh g^−1^ at 1 A g^−1^ after 800 cycles) for LIBs, which are much better than the carbon materials derived from simple carbonization of flour without nickel nitrate.

## 2. Experimental Section

### 2.1. Preparation of Bio-Derived Carbon Materials

Wheat flour was purchased from a supermarket. Typically, 1 g of Ni (NO_3_)_2_·6H_2_O (Aladdin, Shanghai, China) was dispersed into 5 mL deionized water (Aladdin, Shanghai, China) under stirring until dissolved. Then 5 g of wheat flour was added into the above solution gradually to knead repeatedly. The obtained dough was freeze-dried and placed into a porcelain boat, and transferred into a tubular furnace (Kejing Material Technology Co. LTD, Hefei, China). The heating rate was 5 °C min^−1^ from room temperature to 600 or 800 °C in an atmosphere of N_2_ for 2 h. Subsequently, the obtained black monolith was cracked into powders and washed by hydrochloric acid and deionized water for several times. Finally, the NC-600 and NC-800 samples were separated by vacuum filtering and dried under vacuum at 80 °C for 24 h.

Additionally, the WFC-600 and WFC-800 were prepared in the same way as NC-600 and NC-800 without adding Ni(NO_3_)_2_. The numbers “600” and “800” represented the carbonization temperatures.

### 2.2. Analysis and Characterization

The morphology and structure of the obtained NC-600(800) and WFC-600(800) were inspected by an FEI Sirion 200 (Netherlands) high-resolution scanning electron microscope (SEM, FEI, Eindhoven) and a JEOL JEM 2100 (Japan) transmission electron microscope (TEM JEOL, Tokyo, Japan). The X-ray photoelectron spectroscopy (XPS, Escalab 250, Thermo Fisher Scientific, Waltham, MA, USA) was utilized to analyze the surface chemistry. The elements distribution was detected by energy dispersive spectroscopy (EDS, FEI, Eindhoven, The Netherlands). The phase was characterized by X-ray diffraction (XRD, Panalytical X PertPro advance diffraction, Bruker AXS, Karlsruhe, Germany) with Cu Kα radiation. Thermogravimetric analysis (TGA) was carried out using a Perkin-Elmer instrument (SDT 650, TA Instruments, New Castle, DE, USA) at a heating rate of 10 °C min^−1^ ranging from room temperature to 800 °C in the air. Raman spectra (LabRAM HR800 from JY Horiba, Paris, France) was performed to characterize the crystal structure. The specific surface area was investigated by the Barrett-Emmett-Teller (BET) method by an ASAP2460 system. Adsorption in pore size distribution was studied by the nonlocal density functional theory (NLDFT).

### 2.3. Electrochemical Test

The electrochemical properties of the NC-600(800) and WFC-600(800) samples were analyzed by coin-type cells (CR2032, Canrd, Guangdong, China). Typically, the coating slurries were prepared with the active materials, polyvinylidene fluoride (PVDF, ARKEMA, Paris, France), and conducting agent (Super P, Lion, Tokyo, Japan) with a mass ratio of 8:1:1 in N-methyl-2-pyrrolidinone solvent (Aladdin, Shanghai, China). The prepared mixture was coated on a Cu foil and dried under vacuum at 80 °C for 12 h. Then, the Cu foil was cut into discs with a diameter of 1.2 cm, where the mass loading of the active materials for each Cu foil was about 1–2 mg cm^−2^. The Li foil was used as a counter electrode and Celgard 2400 membrane as the separator. About 1M LiPF_6_ dissolved in ethylene carbonate (EC) and diethyl carbonate (DEC) (1:1 by volume) was used as the electrolyte (Fosai, Suzhou, China). The cells 2032 coin were all assembled in an argon-filled glove box. The LAND CT2001A battery instrument (Lanhe, Wuhan, China) was applied to test the Galvanostatic charge/discharge (GCD) cycling with the range of 0.01–3 V at various current rates. CHI660D electrochemical workstation was used to test the cyclic voltammetry (CV) at room temperature. The PARSTAT 4000 electrochemical workstation was applied to analyze the electrochemical impedance spectroscopy (EIS) results at the frequency range of 100 kHz to 0.01 Hz.

## 3. Results and Discussion

The synthesis process of the nickel@carbon materials is as follows. First, a dough is prepared by repeated malaxation of the mixture of wheat flour, nickel nitrate, and deionized water. After freeze-drying, the dried dough is carbonized under N_2_ protection. After the sample was washed by hydrochloric acid and deionized water, the final samples are successfully acquired after drying in the vacuum.

**Figure 1:**
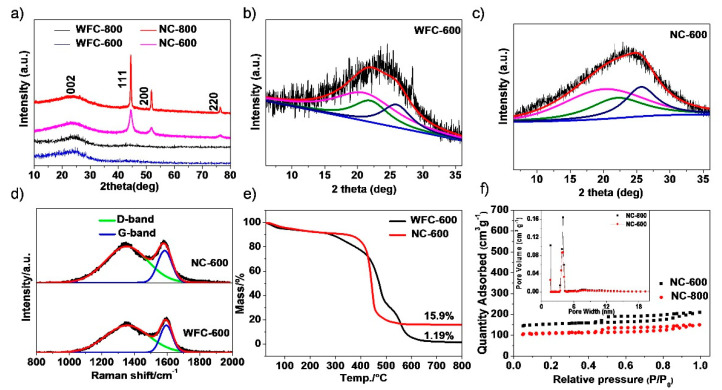
(**a**) XRD patterns of WFC-800, NC-800, WFC-600 and NC-600; (**b**,**c**) the fitting of (002) peaks for WFC-600 and NC-600; (**d**) Raman spectra of WFC-600 and NC-600; (**e**) TG curves of WFC-600 and NC-600; (**f**) N_2_ adsorption−desorption isotherms and the corresponding pore-size distribution curves of NC-600 and NC-800.

As shown in the XRD patterns (Figure 1a), all the four samples exhibit a broad diffraction peak at ~25°, ascribing to the (002) lattice plane of graphitic carbon. As for the prepared NC-600 and NC-800, three sharp diffraction peaks at 2θ = 44.5, 51.8, and 76.4° are observed, which could be assigned to the lattice planes of (111), (200), and (220) for nickel metal with (Fm-3m) space group (JCPDS card 65-2865), respectively [36,37,38,39]. The metallic nickel is formed via a reduction reaction of Ni (NO_3_)_2_ by the surrounding carbon atoms under high carbonization temperature [40,41]. It is well-known that the crystalline structure of the carbon anode materials, for example, the (002) lattice plane, significantly influences the intercalation/de-intercalation of Li^+^. The (002) peaks of the obtained samples are asymmetric, indicating the coexistence of graphitic and amorphous domains [9]. The peak fitting of the (002) lattice plane can be seen in Figure 1b,c and Figure S1, and Table S1. The microspores result from cross-links between smaller domains of graphitic stacks that could increase the average interlayer spacing [8]. According to Table S1, the d-spacing range of the WFC-600, NC-600, WFC-800, and NC-800 above 0.401 nm is ascribed to highly disordered nanocrystallites for Li^+^ insertion freely. The d-spacing of the WFC-600, NC-600, WFC-800, and NC-800 range from 0.379 to 0.398 nm corresponds to “pseudo-graphitic” carbon, and the range from 0.336 to 0.342 nm and is referred to as “graphite-like” carbon [42,43]. Consequently, the Ni^2+^ could assist the chemical modification by promoting the formation of the expanded nanographite, which can supply more intercalation active sites for Li^+^ storage.

Figure 1d and Figure S2 show the Raman spectra of WFC-600, NC-600, WFC-800, and NC-800. Two main peaks at ~1584 cm^−1^ and ~1351 cm^−1^ correspond to the in-plane symmetric sp^2^ C-C bondstretching vibration of graphitic carbon (G band) and the vibration of disordered carbon (D band) at the edges of the graphite sheets, respectively [44]. As is well-known, the intensity ratio of I_D_/I_G_ demonstrates the degree of disorder and the average size of the sp^2^ domains [10,45]. The peak shape of the obtained products is quite similar. The I_D_/I_G_ values (Table S1) of NC-600 and NC-800 are lower than those of WFC-600 and WFC-800, respectively, indicating the sp^2^ content increases and some graphite-like nanocrystalline structure forms in the nickel@carbon materials possibly because of the catalyzation effect of Ni particles [9,46].

Figure 1e shows the TG curves of WFC-600 and NC-600. The small weight loss below 200 °C is on account of the evaporation of the H_2_O molecule which adsorbed on the surface of the sample. There are obvious differences between the WFC-600 and NC-600 in thermogravimetric behavior curves above 200 °C. The WFC-600 and NC-600 exhibit weight loss of 13.9% and 6.6% from 270 °C and 400 °C respectively, indicating the latter sample possesses much higher oxidation resistance performance. The possible reason is that the Ni nanoparticles in NC-600 catalyze the graphitization of the carbon materials during the carbonization process, reflected by the results of XRD and Raman spectra. When the temperature reaches 800 °C, the remaining weight of WFC-600 is only 1.19%, suggesting a tiny content of ashes. Comparatively, the remaining weight of NC-600 is 15.9% (mainly NiO) [47,48]. Accordingly, the Ni content of NC-600 could be determined to be 12.5%. N_2_ sorption analysis was performed to measure the specific surface area and the porosity of the carbon material (Figure 1f). The large adsorption quantities at the very low relative pressure and the small adsorption quantities at p/p^0^ > 0.4 indicate that NC-600 and NC-800 have a dominant microporosity and minor mesoporosity. This is more visible in the pore size distribution plots. The BET surface specific area of NC-600 and NC-800 is 470.5 m^2^ g^−1^ and 321.3 m^2^ g^−1^ (Table 1), respectively. The porous structure is probably formed by the reaction of Ni(NO_3_)_2_ and wheat flour in the carbonization process and the removal of Ni metal during the post-treatment of HCl [11]. Comparatively, the specific surface areas of WFC-600 and WFC-800 are very low indicating a nonporous structure.

**Figure 2:**
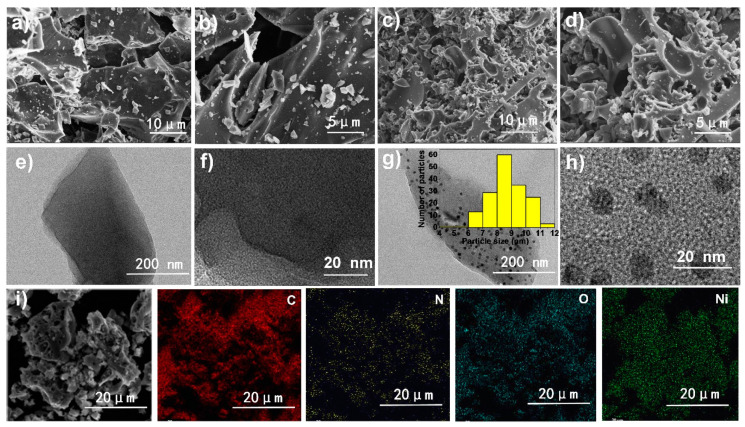
SEM images of (**a**,**b**) WFC-600 and (**c**,**d**) NC-600; TEM of (**e**,**f**) WFC-600 and (**g**,**h**) NC-600; (**i**) EDS mapping of the NC-600.

The morphology and microstructure of WFC-600, NC-600, WFC-800, and NC-800 are illustrated in Figure 2 and Figures S3 and S4. WFC-600 (Figure 2a,b and Figure S3a) exhibits a relatively smooth surface, and no obvious pores are observed on its surface [20]. Comparatively, numerous pores appear on NC-600 (Figure 2c,d and Figure S3b). Similar results are obtained for WFC-800 and NC-800 (Figure S4).

TEM observations demonstrate that the nickel nanoparticles are homogeneously distributed in the NC-600 carbon (Figure 2g,h). The size of Ni particles ranged from 4.7 to 12 nm and approximately 8.7 nm on average determined by the number frequency histogram (inset in Figure 2g). Figure S5d displays that after carbonization at 600 °C, some graphite-like clusters appear on the edges of the Ni nanoparticles with lower interplanar ordering, and most of them in the material are disorder carbon at 600 °C [9]. Figure 2h shows the HR-TEM image of nickel nanoparticles. The fringes with spacings of 0.206 nm correspond to the (111) face of nickel metal. Compared with NC-600, NC-800 (Figure S6) shows an obvious graphitic-like structure and more graphitized carbon fragments are distributed around the Ni particles because of the high carbonization temperature. Therefore, the graphitization degree of NC-800 is significantly enhanced. Moreover, the metal lattice could also be found in NC-800 apparently (Figure S6c,d). The type of elements and their contents of the prepared samples are determined by energy dispersive spectroscopy and are illustrated in Table 1. Moreover, the C, N, and O elements are homogeneously distributed in the WFC-600 (Figure S7) while the C, N, O, and Ni are uniformly observed in NC-600 (Figure 2i), as confirmed by EDS characterization.

**Figure 3:**
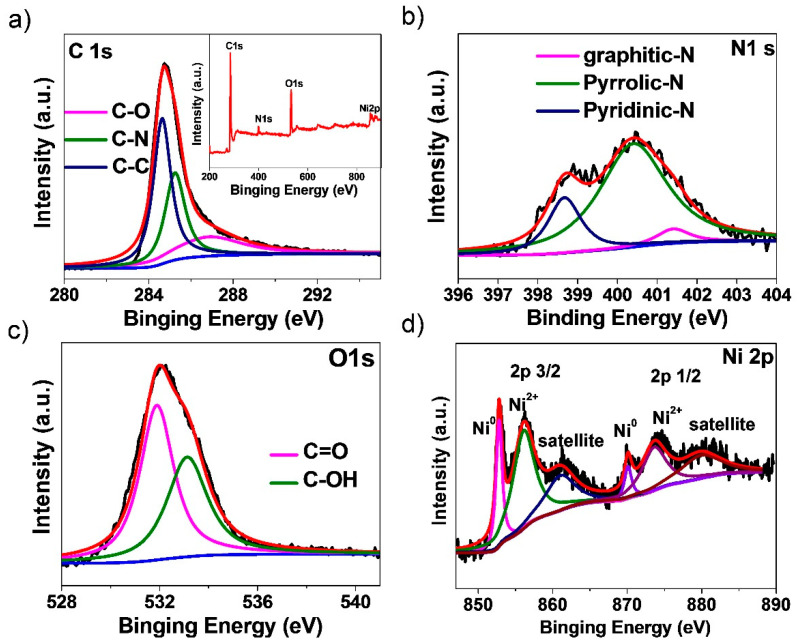
(**a**) XPS spectrum of the NC-600 and fine XPS spectrum of C 1s, (**b**) N 1s (**c**) O 1s, (**d**) Ni 2p spectra for NC-600.

The X-ray photoelectron spectrum (XPS) for NC-600 (inset in Figure 3a) demonstrates that the NC-600 is composed of C, N, O, and Ni elements. In the case of C-species, the spectrum of NC-600 could be deconvolved into three peaks. The peaks at 284.6, 285.2, and 286.4 eV are assigned to the sp^2^ hybridized C-C, C-N, and C-O, respectively. In the case of N-species, pyridinic-N, pyrrolic-N, and graphitic-N of NC-600 are found because the peaks are centered at 398.7, 400.4, and 401.4 eV, respectively (Figure 3b) [49]. In the case of O-species, including the C=O quinone group and C-OH phenol group bonds, the peaks are located at 531.3 and 533.7 eV, respectively (Figure 3c) [50]. The peaks in Ni 2p spectrum (Figure 3d) centered at 852.8 and 870.1 eV are referred to as Ni 2p 3/2 and Ni 2p 1/2 signals of metallic nickel Ni^0^. The peaks at 856.1, 873.6, 861.0, and 879.9 eV are associated with the Ni^2+^, which are generated from the surface partial oxidation of nickel [51]. Additionally, the NiO could not be detected by XRD, demonstrating that the major existence form of nickel is metallic Ni^0^ [52].

**Figure 4:**
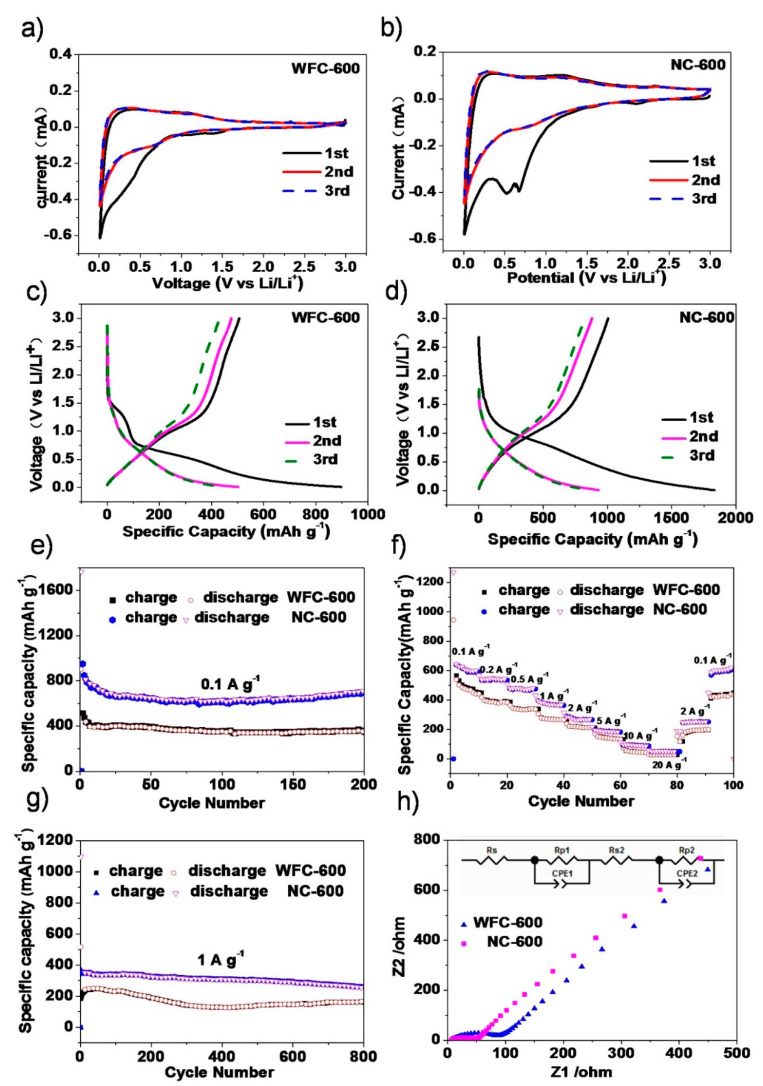
Cyclic voltammetry curves for (**a**) the WFC-600, (**b**) NC-600 at initial three cycles at 0.2 mV s^−1^; the galvanostatic discharge-charge voltage profiles for (**c**) WFC-600, (**d**) NC-600 samples at 0.1 A g^−1^; (**e**) the cyclic performance at 0.1 A g^−1^ for WFC-600 and NC-600; (**f**) the tests of rate-performance of WFC-600 and NC-600; (**g**) the cyclic performance of WFC-600, NC-600 at 1 A g^−1^; (**h**) EIS spectra of WFC-600 and NC-600.

The initial 3 CV curves (Figure 4a,b and Figure S8a,b) investigate the electrochemical properties of the NC-600 (800) and WFC-600 (800) for LIBs. The CV profiles of the WFC-600 and WFC-800 (Figure 4a and Figure S8a) are typical carbonaceous anode materials [53]. In the initial CV curve of NC-600 (Figure 4b), a wide cathodic current peak present at 0.63 V is unavailable in the 2nd and 3rd scans. The reason could be assigned to the formation of SEI (solid electrolyte interface) film, the Li^+^ insertion into carbon material, and the reduction of NiO to metallic Ni [11,51]. During the subsequent anodic scan, the two weak peaks at 0.24 and 1.25 are ascribed to the Li+ extraction from the graphitic carbon layers, the partial decomposition of the SEI layer, and the oxidation reaction of Ni to NiO [8,13]. Moreover, these subsequent CV curves nearly overlap, suggesting that the electrode supports good stability during the lithiation/delithiation process [54]. In the 1st cycle of NC-800 (Figure S8b), there are two obvious cathodic peaks at 0.5 and 1.1 V which could be referred to the reduction of NiO with Li^+^ and to the generation of metallic Ni and Li_2_O, the formation of the SEI layer. Afterward, in the anodic process, two weak anodic peaks occur at 1.6 V and 2.6 V ascribed to the oxidation reaction of Ni to NiO. The two cathodic peaks in the subsequent scan are moved to 0.75 V and 1.5 V because of the modification of the microstructure and the electrolyte decomposition [51]. Moreover, the CV curves of the second and the third cycles are overlapped, suggesting good reversible electrochemical reactions [4,38].

Figure 4c,d and Figure S8c,d delivers the galvanostatic charge/discharge profiles for WFC-600, NC-600, WFC-800, and NC-800 samples at 1st, 2nd, and 3rd cycles at 0.1 A g^−1^. The first coulombic efficiencies of the WFC-600 and NC-600 are 56.4% and 57.6%, respectively, while those of the WFC-800 and NC-800 are only approximately 37.5% and 50%, respectively. The reason probably is the electrolyte decomposition at the electrode/electrolyte interface [55]. Figure 4e depicts the cyclic performance of the prepared samples at 0.1 A g^−1^. The capacity of the NC-600 is 700.8 mAh g^−1^ at the current density of 0.1 A g^−1^ after 200 cycles, better than that of WFC-600 (342.9 mAh g^−1^). A similar result is obtained for NC-800 and WFC-800, with the discharge capacities of 464.8 and 297.4 mAh g^−1^ at 0.1 A g^−1^, respectively (Figure S9). These facts demonstrate the significant positive effect of the Ni embedding on the Li^+^ storage performance. First, the Ni catalyzes the formation of the graphite-like carbon microcrystalline structure, thus providing more stable intercalation sites for Li^+^ [42]. Second, the activation reaction between Ni(NO_3_)_2_ and carbon and the acid-etching post-treatment introduce abundant micropores into the Ni@carbon, leading to additional defect sites for Li^+^ storage and better ionic diffusivity [8,9,56,57]. Additionally, the charge transfer may be improved because of the existence of Ni nanoparticles proved by EIS spectra next.

Figure 4f exhibits the rate property of the WFC-600 and NC-600 at 0.1, 0.2, 0.5, 1, 2, 5, 10, and 20 A g^−1^ for LIBs. The NC-600 can deliver reversible capacities of 609.6, 547.95, 476.4, 369.9, 264.1, 188.6, 92.6, and 51.3 mAh g^−1^ for LIBs, which are higher than those of WFC-600 obtained at the same current densities. The specific capacity of NC-600 remains 51.3 mAh g^−1^ at 20 A g^−1^, and then the capacity quickly recovered when the current density reduced to 0.1 A g^−1^, demonstrating good charge-discharge transfer kinetics within the NC-600 architecture. The specific charge capacities of NC-800 (Figure S9) are lower than those of NC-600 because of the higher content of graphitic fragments for NC-800 limits the insertion of Li^+^. WFC-600 and NC-600 exhibit good long cycle performance at 1A g^−1^ for LIB. After 800 cycles, the capacity of WFC-600 and NC-600 is 162.8 and 257.25 mAh g^−1^, respectively (Figure 4g). Previously reported biomass-derived carbons and metal@carbon materials are sweet potato-derived carbon (320 mAh g^−1^ at 0.1 A g^−1^ after 200 cycles), NiO/C hollow microspheres (628 mAh g^−1^ at 0.1 A g^−1^ after 100 cycles), C/Ni700 (205 mAh g^−1^ at 1C after 50 cycles), NiO-graphene (646 mAh g^−1^ at 0.1 A g^−1^ after 35 cycles) and Ni-graphene (675 mAh g^−1^ at 0.1 A g^−1^ after 35 cycles), etc., (Table 2). Comparatively, NC-600 exhibits the advantages in rate capacity (51.3 mAh g^−1^ at 20 A g^−1^) and cycling stability (700.8 mAh g^−1^ at 0.1 A g^−1^ after 200 cycles; 257.2 mAh g^−1^ at 1 A g^−1^ after 800 cycles). The unique structure of the NC-600 with nanocrystalline structure and the introduced defect sites lead to good Li^+^ storage and better ionic diffusivity. Therefore, NC-600 is a material with high performance and good application prospects.

The electronic transport properties of the materials are explored by the electrochemical impedance spectra (Figure 4h and Figure S9c). Typically, the straight line is ascribed to the ion diffusion process (R_w_), the following semicircle is associated with the charge transfer resistance (R_ct_), and the high-frequency region reflects the electrode resistance (R_e_). The lower charge transfer resistance of NC-600 (NC-800) than WFC-600 (WFC-800) is because the microstructure of the former electrode materials with more defects can provide additional sites, and the anchored nickel nanoparticles can initiate fast electron transfer and better ionic diffusivity during the cycling process [7,8,23,58].

**Figure 5:**
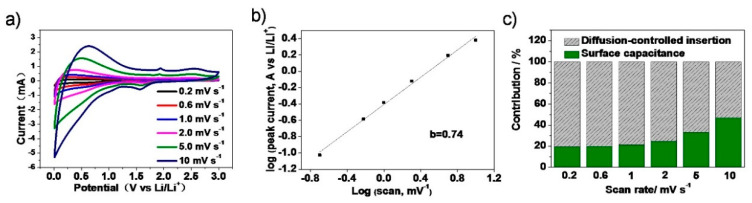
Cyclic voltammograms of NC-600 at various scan rates (**a**); the relationship between peak currents and scan rates (**b**); survey of the capacitive and diffusion-controlled contribution ratio at different scan rates; Normalized contribution ratio of diffusion and capacitive capacities at different scan rates of NC-600 (**c**).

The CV profiles of NC-600 at various scan rates from 0.2–10 mV s^−1^ are shown to investigate the contributions of different mechanisms for LIBs (Figure 5a). The Equations (1) and (2) can express the charge storage mechanism between the response currents (*i*) and the scan rates (*v*) [44].

(1) $$i=av^{b}$$

(2) $$i(V)=k_{1}v+k_{2}v^{0.5}$$

Here, *b* is an indicator of capacitive process free of diffusion limitations (when *b* = 1), diffusion-controlled process (when *b* = 0.5), or mixed mechanism (0.5 < *b* < 1). The *b* value could be obtained from the slope of the log (*i*) versus log (*v*) plots. For carbon anode materials, the diffusion-controlled reaction suggests the intercalation reactions while the capacitive process free of diffusion limitations indicates the electrochemical double layer (EDL) and pseudocapacitive storage processes [65,66,67]. The *b* values for NC-600 is 0.74 (Figure 5b), suggesting a surface capacitive process domination, a combination of diffusion capacitance and surface capacitance lithium storage mechanism. The capacitive contribution ratios gradually grow with the increase of scan rates, reaching up to 46.1% for NC-600 (Figure 5c) at the scan rate of 10 mV s^−1^. The *b* values of NC-600 indicate the good distribution of the main intercalation mechanism for Li^+^ storage which also identifies the formation of the graphite-like carbon structure.

## 4. Conclusions

In summary, we produced a novel hard carbon embedded with nickel nanoparticles (nickel@carbon) by carbonizing the dough which is prepared by mixing wheat flour and the nickel nitrate. The introduced Ni is proved to play a vital role in the catalyzed graphitization and regulated the carbon nanocrystalline structure, and made a positive effect on the Li^+^ storage performance. Because of the unique structure, the NC-600 sample shows the favorable electrochemical performance, with a reversible capacity of up to 700.8 mAh g^−1^ at 0.1 A g^−1^ after 200 cycles, long-term cyclic performance of up to 257.25 mAh g^−1^ after 800 cycles, and good rate capability with 51.3 mAh g^−1^ at 20 A g^−1^. The unique inexpensive NC-600 and their environment-friendly synthesis approach can be applied to develop advanced transition metal/oxides-embed hard carbon materials for future electrochemical energy storage and conversion applications.

## Figures and Tables

**Table 1:** Texture properties and element compositions of the samples.

Sample	Porosity Parameters				Element Composition (atom %)			
	S_BET_ (m^2^g^−1^)	V_T_ (cm^3^g^−1^)	V_meso_ (cm^3^g^−1^)	V_micro_ (cm^3^g^−1^)	C	N	O	Ni
WFC-600	3.3	/	/	/	87.0	4.2	8.8	/
NC-600	470.5	0.3	0.1	0.2	85.1	3.1	9.2	2.6
WFC-800	3.5	/	/	/	84.1	9.7	6.2	/
NC-800	321.3	0.2	0.08	0.1	83.4	2.9	9.7	2.7

**Table 2:** The comparison of LIB properties between the NC-600 and other biomass carbon.

Materials	Specific Capacity (mAh g^−1^)	Current Density (A g^−1^)	Cycle Stability	References
glucosamine-based porous carbon	264	0.1	100	[11]
structurally tunable carbon	217	1C	100	[28]
nano-porous hard carbons	453.1	0.2C	50	[59]
nitrogen-doped porous hollow carbon	512	1.5C	500	[46]
sweet potato-derived carbon	320	0.1	200	[13]
N-doped graphitized hard carbon	389	0.1	100	[60]
rice husk-derived carbon	403	0.2C	100	[61]
Loofah-derived carbon	225	0.1	200	[62]
NiO/C hollow microspheres	628	0.1	100	[54]
Ni-graphene	675	0.1	35	[58]
NiO-graphene	646.1	0.1	35	[63]
C/Ni700	205	1C	200	[64]
NC-600	700.8	0.1	200	this work
	257.2	1	800	
	51.3	20	10	

## Supplementary Materials

The following are available online at https://www.mdpi.com/1996-1944/13/20/4611/s1, Figure S1: Peak fitting of the (002) peaks of (a) WFC-800 and (b) NC-800; Figure S2. (a) Raman spectrum; (b,c) Raman spectra of WFC-800 and NC-800; Figure S3. SEM images of (a) WFC-600 and (b) NC-600; Figure S4. (a–c) SEM images of WFC-800 and (d–f) NC-800; Figure S5. TEM images of (a,b) WFC-600 and (c,d) NC-600; Figure S6. TEM images of (a,b) WFC-800 and (c,d) NC-800; Figure S7. EDS of the WFC-600; Figure S8. Cyclic voltammetry curves of (a) the WFC-800, (b) NC-800 at the first three cycles; the charge-discharge profiles of (c) WFC-800 and (d) NC-800 samples at initial three cycles at 0.1 A g^−1^; Figure S9. (a) The cyclic performance of WFC-800 and NC-800 at 0.1 A g^−1^; (b) the rate-performance of WFC-800 and NC-800 electrodes; (c) EIS spectra of WFC-800 and NC-800 for LIBs, Table S1. Physical parameters of the composites from XRD and Raman patterns.
