# Supplementary material for: Nickel-Embedded Carbon Materials Derived from Wheat Flour for Li-Ion Storage
Source: Materials (Basel). 2020 Oct 16;13(20):4611. doi: 10.3390/ma13204611 (PMC7602715; doi:10.3390/ma13204611)
Supplement: Supplementary file 1 [file materials-13-04611-s001.pdf]

materials

Supporting Information

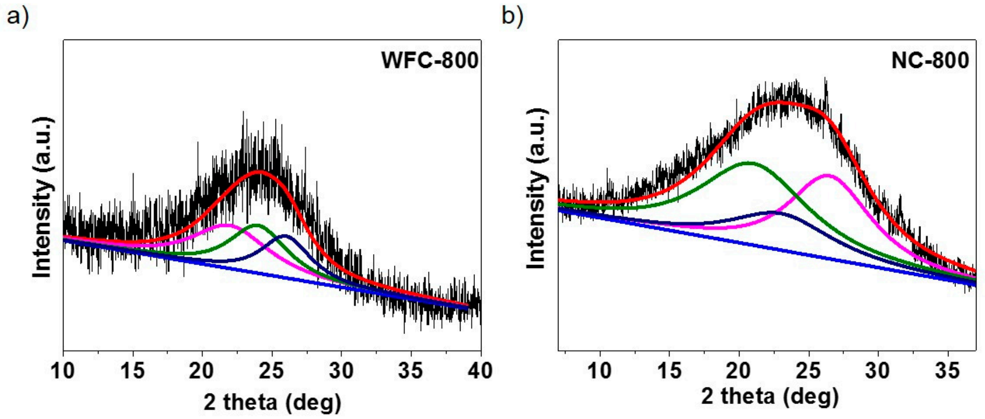

Figure S1. Peak fitting of the (002) peaks of (**a**) WFC-800 and (**b**) NC-800.

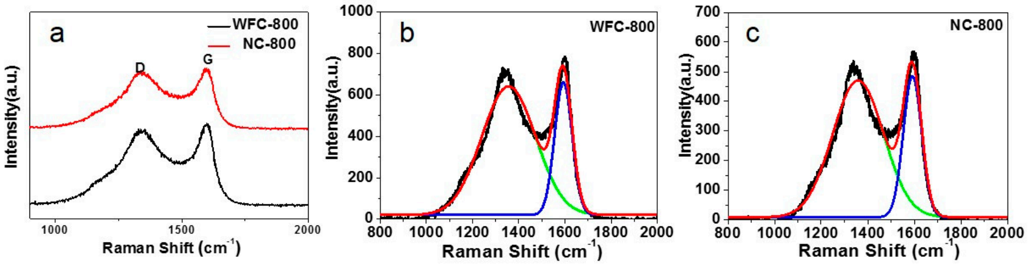

Figure S2. (**a**) Raman spectrum; (**b**,**c**) Raman spectra of WFC-800 and NC-800.

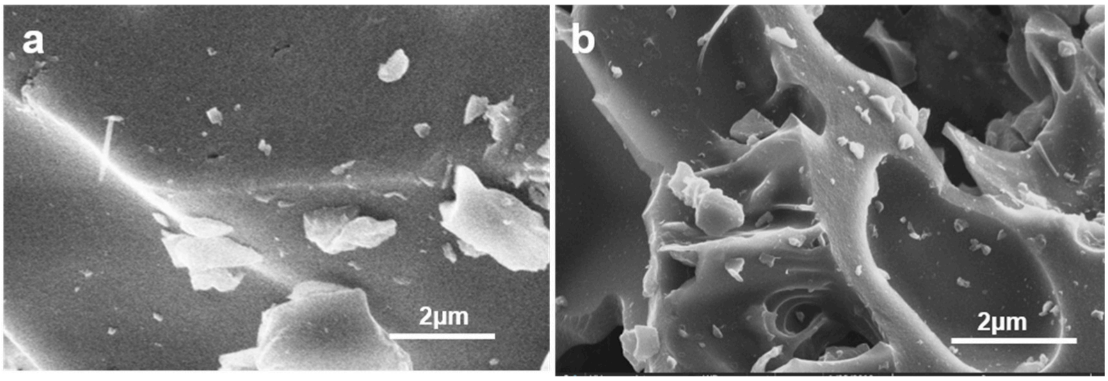

Figure S3. SEM images of (**a**) WFC-600 and (**b**) NC-600.

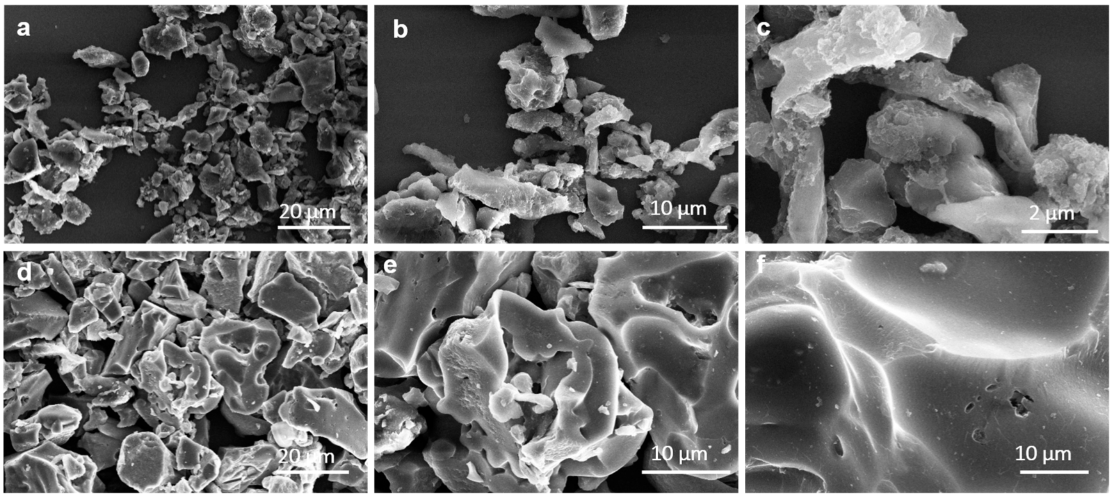

Figure S4. (**a**–**c**) SEM images of WFC-800 and (**d**–**f**) NC-800.

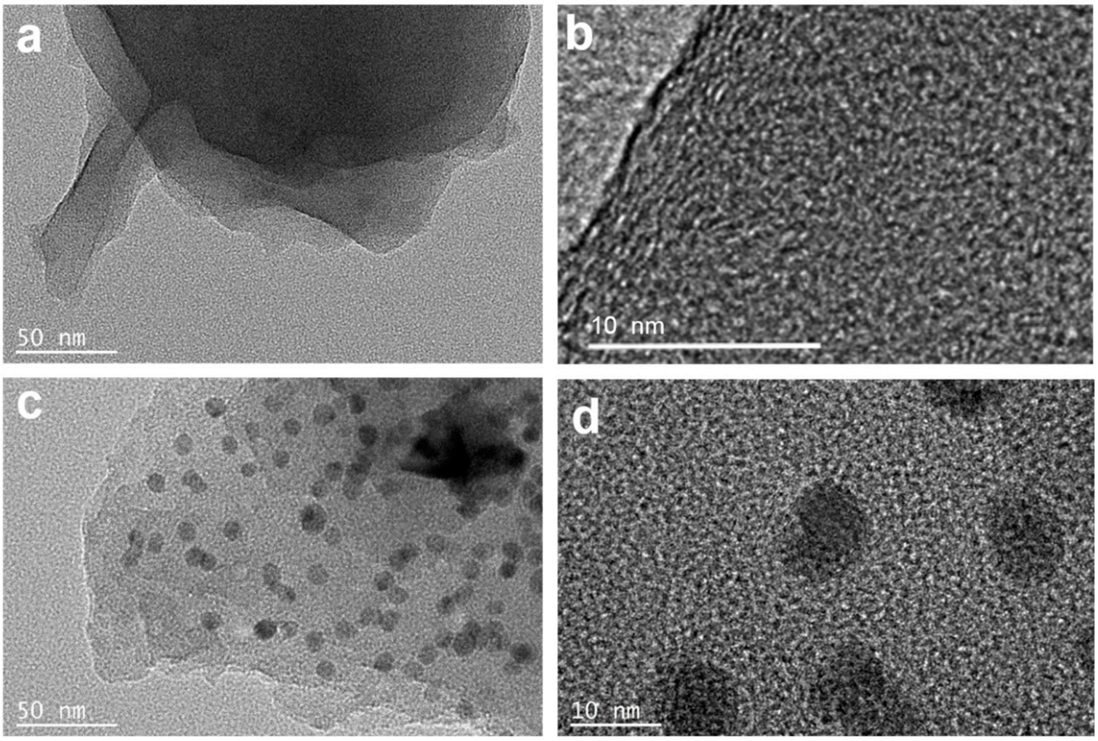

Figure S5. TEM images of (**a**,**b**) WFC-600 and (**c**,**d**) NC-600.

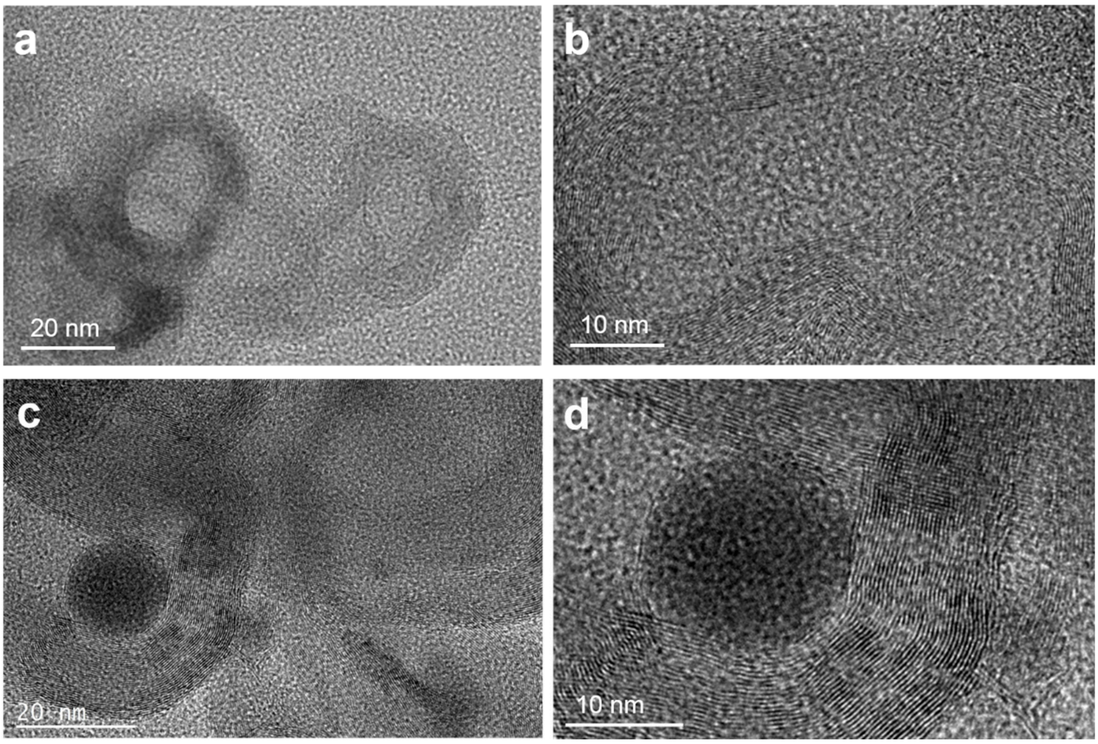

Figure S6. TEM images of (**a**,**b**) WFC-800 and (**c**,**d**) NC-800.

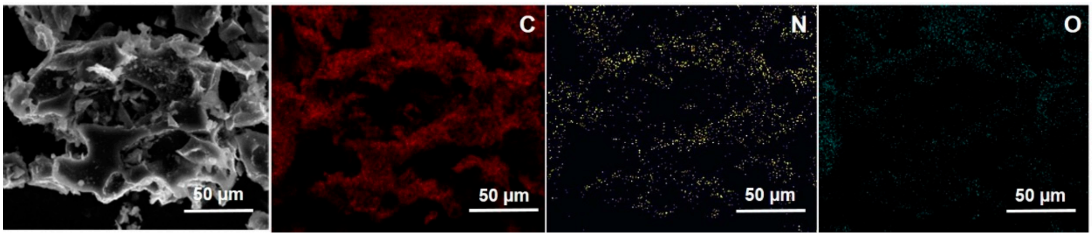

Figure S7. EDS of the WFC-600.

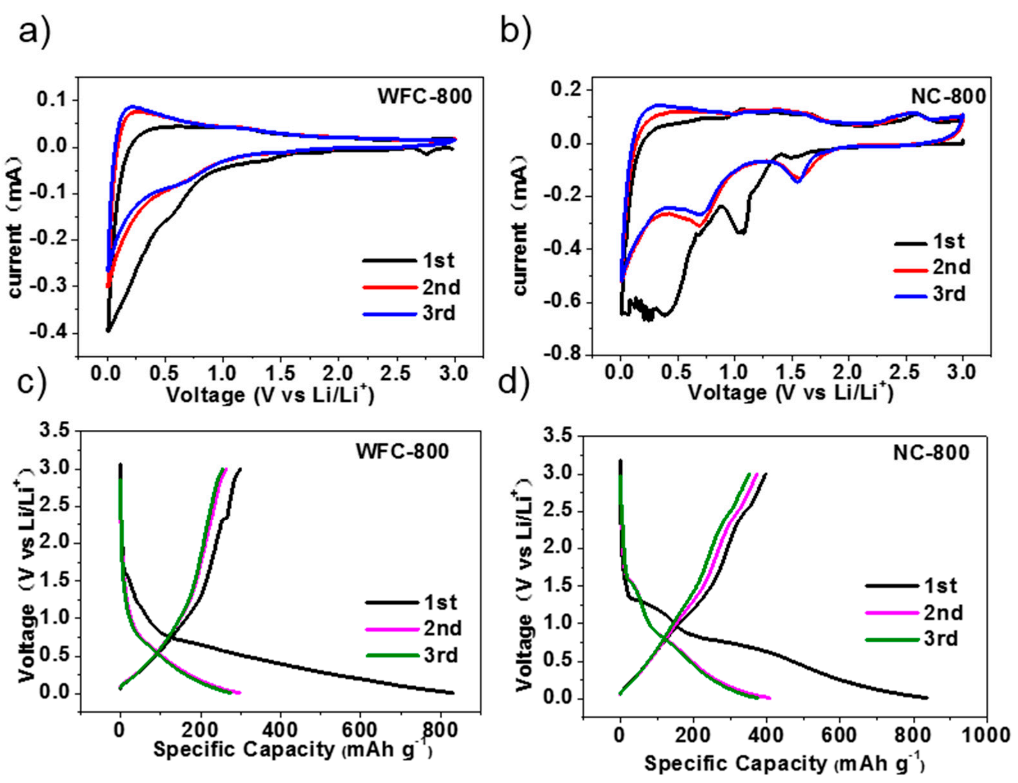

Figure S8. Cyclic voltammetry curves of (**a**) the WFC-800, (**b**) NC-800 at the first three cycles; the charge-discharge profiles of (**c**) WFC-800 and (**d**) NC-800 samples at initial three cycles at 0.1 A g^{-1}.

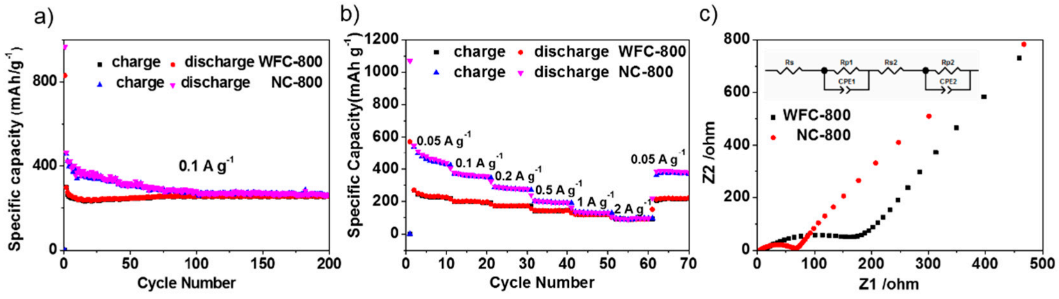

Figure S9. (**a**) The cyclic performance of WFC-800 and NC-800 at 0.1 A g^{-1}; (**b**) the rate-performance of WFC-800 and NC-800 electrodes; (**c**) EIS spectra of WFC-800 and NC-800 for LIBs.

Table S1. Physical parameters of the composites from XRD and Raman patterns.

Sample	High disordered			Pseudo-graphitic			Graphite-like			
	2θ	d_{002}	Area(%)	2θ	d_{002}	Area(%)	2θ	d_{002}	Area(%)	I_D/I_G
WFC-600	21.03	0.423	55.0	22.34	0.398	24.0	26.05	0.342	21.0	2.62
NC-600	21.24	0.418	47.0	22.51	0.394	28.1	26.13	0.341	25.0	1.88
WFC-800	22.32	0.396	40.6	23.29	0.381	33.9	26.31	0.338	25.5	1.59
NC-800	22.12	0.383	45.7	23.41	0.379	19.0	26.50	0.336	35.3	1.29
